# Helminth Colonization Is Associated with Increased Diversity of the Gut Microbiota

**DOI:** 10.1371/journal.pntd.0002880

**Published:** 2014-05-22

**Authors:** Soo Ching Lee, Mei San Tang, Yvonne A. L. Lim, Seow Huey Choy, Zachary D. Kurtz, Laura M. Cox, Uma Mahesh Gundra, Ilseung Cho, Richard Bonneau, Martin J. Blaser, Kek Heng Chua, P'ng Loke

**Affiliations:** 1 Department of Parasitology, Faculty of Medicine, University of Malaya, Kuala Lumpur, Malaysia; 2 Jeffrey Cheah School of Medicine and Health Sciences, Monash University Malaysia, Johor Bahru, Johor, Malaysia; 3 Department of Medicine, New York University School of Medicine, New York, New York, United States of America; 4 Department of Microbiology, New York University School of Medicine, New York, New York, United States of America; 5 Department of Biology, Center for Genomics and Systems Biology, New York University, New York, New York, United States of America; 6 Department of Biomedical Science, Faculty of Medicine, University of Malaya, Kuala Lumpur, Malaysia; Uniformed Services University, United States of America

## Abstract

Soil-transmitted helminths colonize more than 1.5 billion people worldwide, yet little is known about how they interact with bacterial communities in the gut microbiota. Differences in the gut microbiota between individuals living in developed and developing countries may be partly due to the presence of helminths, since they predominantly infect individuals from developing countries, such as the indigenous communities in Malaysia we examine in this work. We compared the composition and diversity of bacterial communities from the fecal microbiota of 51 people from two villages in Malaysia, of which 36 (70.6%) were infected by helminths. The 16S rRNA V4 region was sequenced at an average of nineteen thousand sequences per samples. Helminth-colonized individuals had greater species richness and number of observed OTUs with enrichment of Paraprevotellaceae, especially with *Trichuris* infection. We developed a new approach of combining centered log-ratio (clr) transformation for OTU relative abundances with sparse Partial Least Squares Discriminant Analysis (sPLS-DA) to enable more robust predictions of OTU interrelationships. These results suggest that helminths may have an impact on the diversity, bacterial community structure and function of the gut microbiota.

## Author Summary

Soil-transmitted helminths are carried by large numbers of people in developing countries. These parasites live in the gut and may interact with bacterial communities in the gut, also called the gut microbiota. To determine whether there are alterations to the gut microbiota that are associated with helminth infections, we examined the types of bacteria present in fecal samples from rural Malaysians, many of whom are helminth-positive and find it likely that helminth colonization alters the gut microbiota for rural Malaysians.

## Introduction

Today, approximately a quarter of the world's population carries soil-transmitted helminths [1]. In an endemic tropical environment, more than 70% of the population may be infected with helminths [2], which is indicative of our helminth exposure during the course of human evolution. Just as the commensal microbiota has coevolved with mammalian hosts [3], they must also have coevolved with helminths within their mutual hosts [4]. However, there is little understanding of how the presence of helminths may affect the microbial ecology of the human gut. While the healthy human microbiota from individuals of the developed world is well-characterized [5], residents of developing countries harbor very different bacterial communities on the skin [6], and in the gut [7], with an increased abundance of the genus *Prevotella* [8]. While diet has been assumed to contribute most significantly to the differences of the intestinal tract, it is possible that helminth infections also may have a substantial impact on the human microbiome [9].

Alterations in the human microbiome have been associated with a range of conditions in the developed world, including inflammatory bowel disease (IBD) [10]–[12], obesity [13]–[15] autism [9], type 2 diabetes [16] and allergies [17]–[19]. These diseased states have often been linked with decreased diversity of the microbiota, perhaps because healthy species-rich communities are more stable and resistant to pathogenic invasions [20]. Acute gastrointestinal infections also are associated with reductions in microbial diversity and increased volatility of microbial communities [21]. In contrast, helminth exposure has been shown to restore microbial diversity to macaques suffering from idiopathic chronic diarrhea [22]. While recent studies have begun to establish changes to microbial communities in response to intestinal helminths [4], [23], most studies to date have been on animal models and not on human subjects.

As Malaysia experiences rapid development, helminth infections have generally decreased, however they are still highly prevalent among the Malaysian indigenous populations [24], who live in the rural and semi-urban areas of Peninsular Malaysia. The aim of our study was to compare the gut microbiota of helminth-infected individuals within this community, with individuals who were not colonized by helminths. Based on our previous studies with macaques experimentally infected with helminths [22], we hypothesized that helminth infected individuals may have increased microbial diversity relative to uninfected individuals.

Genomic surveys of the microbiota require the development of new approaches to examine the interactions of microbes with each other and their hosts. The abundance of each microbial operational taxonomic unit (OTU – i.e. the best known taxonomic specification of a population as measured by high throughput sequencing of the bacterial 16S gene) reflects the product of the total microbial community size times the relative fitness of that OTU's population in the measured community/environment. Ecological relationships should be detectable via similarity measures (e.g. - correlations) between microbial abundances and host variables (e.g. helminth infection status). However, compositional artifacts are a major confounder hindering functional microbial inference with standard statistical approaches and requiring the development of new methods. These compositional artifacts are a result of the fact that community total size varies and that ‘components’/OTU abundances are measured as relative proportions of this unknown total community size (for example in this study OTU abundances for each sample must sum to 1 in a typical microbiome datasets in order to normalize for sampling or sequencing depth). Another common problem with the analysis of these datasets is that the number of variables of interest is usually much larger than the number of available samples, and thus we are typically undersampled with respect to analysis that aim to detect relationships between microbes in complex communities. In order to address these two problems for our dataset, we devised a strategy to combine centered log-ratio (clr) transformation [25] for OTU relative abundances with sparse Partial Least Squares Discriminant Analysis (sPLS-DA) [26] in order to identify OTU features that are more predictive of helminth infection status. This approach is general and could also be easily applied to other microbiome studies in the future. R source code for our analysis can be found at (bonneaulab.bio.nyu/).

Sequencing 16S ribosomal RNA may reveal alterations to taxonomic groups and species composition but it does not provide data on metabolic activity and function of the microbial communities that may be altered by helminth colonization. Microbial function in communities can be assessed using shotgun metagenomics [7], [27], but this approach is expensive and challenging to analyze. PICRUSt is an approach for inferring the metagenome of the closest available whole genome sequences using 16S gene sequence profiles [28], [29], hence it provides a way to predict changes to microbial function likely to be associated with changes in OTU-abundance detected via 16S-sequencing. In this study, we used PICRUSt [28] to investigate functional differences in the microbiota of helminth colonized individuals.

## Methods

### Ethics statement

The Medical Ethics Committee of University Malaya Medical Center (UMMC), Kuala Lumpur, Malaysia under the MEC reference number 943.14, approved the study on indigenous Malaysians. All the adult participants provided written informed consent. Written informed consent was obtained from legal representatives of the children that participated in the study. New York Harbor Veterans Affairs Hospital Institutional Review Board approval was obtained before enrolling subjects in the study in New York. Written informed consent was received from all the participants.

### Study area and sample population

The 51 volunteers (ages 0.4–48 years) from the indigenous community in Malaysia, also known as *Orang Asli* in local terms, lived in two villages of Kampung Ulu Kelaka (N 3° 6′ 0″, E 102° 4′ 1″) (n = 22) and Kampung Dusun Kubur (N 2° 56′ 22.6″, E 102° 4′ 32.3″) (n = 29) that were located in the Jelebu district, Negeri Sembilan state, approximately 80 km east of Kuala Lumpur, capital of Malaysia. Both communities were of the *Temuan* ethnic subgroup, with the majority of the villagers living below the poverty line (i.e., RM 800 or USD 256 per month). The main economic activities are rubber tapping, farming and collecting forest products. Most of the houses in Kampung Ulu Kelaka were built under the government-coordinated Housing Aid Program (PBR) and were equipped with basic amenities such as piped water, toilets and had electric supply. However, in Kampung Dusun Kubur, the villagers were still living in wooden or partial brick houses, without piped water and toilets. While increasingly some of the younger villagers have been able to complete high school education, most of the adult villagers from these two communities have only attended elementary school. Sample collection was carried out in September 2012. The research subjects from New York were recruited at the Manhattan Veteran Affairs Hospital and were all over the age of 50. They were at an average risk for colon cancer and presented for screening colonoscopy. The median age was 60.5. Ethnicity was 30% (n = 6) Caucasian, 25% (n = 5) African American, 30% (n = 6) Hispanic and 15% (n = 3) Other.

### Fecal sample collection, parasitological analysis and DNA extraction

Stool samples with 2.5% potassium dichromate as a preservative were collected in screw-capped containers and stored in 4**°**C for several days prior to DNA extraction. DNA was extracted from the 51 fecal samples using MACHEREY-NAGEL NucleoSpin Soil kit (MACHEREY-NAGEL GmbH & Co. KG, Düren, Germany). The SL 2 buffer was used with 150 µl Enhancer SX in the sample lysis step. DNA was eluted in 50 µl of Buffer SE and stored at −20°C.

The formalin-ether concentration technique was used for microscopic examination. One to two gram(s) of stool sample was mixed with 7 ml of 10% formalin and 3 ml of ethyl acetate in 15 ml falcon tubes. The samples were then centrifuged at 2500 rpm for 5 min. Fecal smears were made, stained with 0.85% iodine and observed by light microscope under the magnification of 100× for helminths (*Trichuris* spp., *Ascaris* spp., and hookworm). Each subject was scored for presence or absence of each of these three helminth species.

### Amplification and sequencing of variable 4 (V4) region of 16S ribosomal RNA (rRNA) genes

V4 rRNA paired-end sequencing was performed on the DNA from the fecal samples collected using the protocol modified from Caporaso et al [30]. The forward primer construct (5′- AAT GAT ACG GCG ACC ACC GAG ATC TAC ACT ATG GTA ATT GTG TGC CAG CMG CCG CGG TAA -3′) contained a 5′ Illumina adapter, a forward primer pad, the 515F primer and a two-base linker sequence (‘GT’). The reverse primer (5′- CAA GCA GAA GAC GGC ATA CGA GAT NNN NNN NNN NNN AGT CAG TCA GCC GGA CTA CHV GGG TWT CTA AT -3′) contained the 3′ Illumina adapter, a unique 12-base error-correcting Golay barcode, the reverse primer pad, a two-base linker sequence (‘CC’) and the 806R primer. Polymerase chain reaction (PCR) was carried out in triplicate using the Bio-Rad CFX 96 system (Bio-Rad, Hercules CA, USA). The PCR mix contained 0.2 µM forward and reverse primers, 1 µl template DNA, 10 µl 5 Prime Hot Master Mix (5 PRIME, Gaithersburg MD, USA) and 12 µl of MoBio PCR certified water (MO BIO Laboratories, Calsbad CA, USA). Thermal cycling consisted of 94°C for 3 min, followed by 35 cycles of 94°C for 45 s, 50°C for 60 s and 72°C for 90 s, with a final extension of 10 min at 72°C to confirm full amplification.

Replicate amplicons were pooled and the DNA concentrations were determined using the Quant-iT PicoGreen dsDNA reagent and kit (Invitrogen, Grand Island NY, USA) based on the manufacturer's instructions. Fluorescence was measured on the Perkin-Elmer Victor Plate reader using the 490/535 nm excitation/emission filter pair with measurement time 0.1 s. The amplicons were then pooled in equimolar ratios and purified using QIAquick PCR purification kit (Qiagen Inc, Chatsworth, CA, USA). The final concentration of cleaned DNA amplicon was determined using the Qubit PicoGreen dsDNA BR assay kit (Invitrogen, Grand Island, NY, USA). Amplicon sequencing was performed on the Illumina MiSeq system (Illumina, San Diego CA, USA).

### Concatenation and quality filtering of Illumina MiSeq reads

We first improved the joining potential of the paired-end raw sequences by trimming low quality bases at the overlapping ends of reads. We used EA-utils [31], [32] to trim and join reads by iterating over a range of Phred quality scores (1–20) as the threshold for terminal base removal. We then joined paired-end reads at each quality score, only accepting the sequence set that resulted in the maximum number of joined sequences and then removed unjoined barcodes. Finally, we proceeded with processing of sequence reads using the Quantitative Insights Into Microbial Ecology (QIIME) software package [33] to filter sequence read quality (minimum quality score of 25, minimum/maximum length of 200/1000, no ambiguous bases allowed and no mismatches allowed in the primer sequence) and to split multiplexed libraries.

### Analysis of quality filtered reads

To identify and quantitate abundances of Operational Taxonomic Units (OTUs) from the sequence data, we used a combination of reference-based and de novo sequence clustering (pick_subsampled_reference_otus_through_otu_table.py). For closed reference-based picking, we aligned sequences to the Greengenes 12_10 reference collection (available at http://greengenes.secondgenome.com/downloads). 0.1% of the sequences that failed to align to the reference were randomly subsampled and clustered de novo using UCLUST [34], with an OTU cluster defined at a sequence similarity of 97%. The centroid sequence of each cluster was chosen as the new reference set for another round of closed-reference OTU picking. OTU assignments for reads that failed to align to this reference collection were picked by another round of de novo clustering. We performed all closed-reference picking by dividing the task into 20 jobs and ran the alignment in parallel on a high performance computing cluster environment. For de novo OTU clusters, representative sequences were picked for taxonomic identity assignment using the Ribosomal Database Project (RDP) classifier [35], [36]. The taxonomy assignment for each sequence was truncated at the most specific taxonomic level with a confidence score of at least 0.8. The PyNAST alignment algorithm [37] was used to align the OTU representative sequences against the Greengenes core database set with a minimum alignment length of 189 and a minimum identity of 75% and FastTree [38] was used to construct a phylogenetic tree. We then generated a final OTU table for downstream analysis by excluding the sequences that had failed to align by PyNAST.

To compare the sequencing data from the Malaysian and New York samples, we preprocessed, quality-filtered and split the libraries of the sequence reads from the two separate MiSeq runs, independently. The files containing the resulting sequence data were concatenated into a single file and a new mapping file with combined sample data were taken together for OTU picking and downstream analysis.

### 16S rRNA gene sequence diversity analysis

Samples were evaluated for beta diversity (community diversity divergence between samples) and alpha diversity (microbial diversity within samples) calculations in QIIME. Beta diversity was calculated using the QIIME default beta diversity metrics for weighted and unweighted UniFrac distances on both uneven and evenly subsampled OTU tables. UniFrac is a measure of the amount of evolutionary history that is unique between samples of at least two different environments [39], [40]. Unweighted UniFrac makes comparisons based on the presence and absence of members, while the weighted version also incorporates abundance information. To identify environments that could drive groupings of similar communities, principle coordinate analysis (PCoA) was performed on the UniFrac distance matrices generated from beta diversity calculation and the resulting PCoA plots were visualized using the KiNG graphics program (http://kinemage.biochem.duke.edu/index.php). Environments producing distinct clustering of samples were subjected to significance testing using the non-parametric statistical analysis ANOSIM via QIIME. Alpha rarefaction was performed using the phylogenetic distance [41] and Shannon index [42] metrics. We rarefied OTU tables so that all sample sizes matched the minimum sampling depth, then randomly subsampled sequences over a range of specified depths and calculated the alpha diversity for each sample at each point, with 10 independent iterations at each depth. We assessed statistical significance of between-group alpha diversity metrics by two-sample t-test as implemented in QIIME.

### LDA Effect Size (LEfSe) analysis

To identify taxa with differentiating abundance in the different environments, the LDA Effect Size (LEfSe) algorithm was used with the online interface Galaxy (http://huttenhower.sph.harvard.edu/galaxy/root). Helminth infection status and country were assigned as the respective comparison classes in two separate analyses (one comparing countries and one comparing helminth status within Malaysia. LEfSe first identified features that were statistically different among the different biological classes. It then performed non-parametric factorial Kruskal-Wallis (KW) sum-rank test and Linear Discriminant Analysis (LDA) to determine whether these features are consistent with respect to the expected behavior of the different biological classes [43].

### Centered log-ratio (clr) transformation and sparse Partial Least Squares Discriminant Analysis (sPLS-DA)

To generate better predictive models of Helminth-microbiome component interactions, we used centered log-ratio (clr) transformations of the relative abundance data (to circumvent the compositional bias problem), in combination with sparse Partial Least Squares Discriminant Analysis (sPLS-DA) (to address the “noise” problem), in order to generate more compositionally robust, predictive models of OTU features. Partial Least Squares discriminant analysis (PLSDA) is a technique to predict a discrete ‘response’ (e.g. - parasite infection status) by regressing on many sample features (e.g. OTU abundances), and finding the set of latent, orthogonal factors that maximizes the covariance between predictors and response variables [26]. It has been used previously on microbiome relative abundances, for example, to identify OTUs associated with antibiotic treatment [44]. However, PLSDA, along with other statistical methods, suffers from problems of compositional artifacts due to unit-sum constraint of relative abundance data.

To account for these compositional artifacts, we first transformed relative abundances using the centered log-ratio (clr) transformation. This maps compositional data to a corresponding Euclidean space by dividing each component by the geometric mean of all the components in a sample and then taking the log of that ratio [25]. Typically, this transformation results in data singularity, which is a major disadvantage for covariance-based techniques. However, this is not a problem for PLSDA, which can efficiently deal with multi-colinearity that can result from the clr transformation [45], [46].

Using a combination of custom R scripts and the *caret* and *spls* packages [47], [48], we clr transformed the data and filtered the OTU table to the set of highly variable OTUs. We performed feature selection using sparse PLS-DA and 5-fold cross validation to tune algorithm parameters (sparsity and number of latent components) and to check model validity. To assess the statistical significance of a feature's contribution to the model, we obtained bootstrapped and null (by randomly permuting data) estimates of PLS-DA coefficients and report the p-value of an OTU as the fraction of bootstrapped coefficients lying at or above the tails of the null distribution. We visualized the resulting models by projecting the clr-transformed data points onto the PLS loadings of sparse PLS-DA and permutation-selected features and visualize these projections as biplots. Code used to perform this analysis is available at bonneaulab.bio.nyu.edu.

### Inferred metagenomics by PICRUSt

The demultiplexed data files from the processed sequencing reads were subjected to another round of closed-reference OTU picking for use in PICRUSt. This was done by aligning the sequences against the newest Greengenes reference OTUs (downloaded from http://greengenes.secondgenome.com/downloads/database/13_5) and OTUs were assigned at 97% identity. The resulting OTU table was then used for microbial community metagenome prediction with PICRUSt on the online Galaxy interface. PICRUSt was used to derive relative Kyoto Encyclopedia of Genes and Genomes (KEGG) Pathway abundance [28]. Supervised analysis was done using LEfSe to elicit the microbial functional pathways that were differentially expressed in individuals with different helminth infection status.

## Results

### Characteristics of the study populations

We compared the stool microbiota of 51 residents from two villages in Malaysia collected on a single field trip (**Table 1**). The majority (n = 36; 70.6%) of the 51 fecal samples collected from the Malaysian indigenous communities contained helminths, with *Trichuris* spp. (n = 28; 54.9%) being the most common, followed by *Ascaris* spp. (n = 21; 41.2%) and hookworm (n = 5; 9.8%). Hence, individuals were often colonized by more than one helminth (n = 17; 33.3%).

**Table 1:** Baseline characteristics of the Malaysian indigenous study participants (N = 51).

Variables	N	%	95% CI
**Age (years)**			
≤12	30	58.8	45.3–72.3
>12	21	41.2	27.7–54.7
**Gender**			
Female	24	47.1	33.4–60.8
Male	27	52.9	39.2–66.6
**Prevalence of helminth infections**			
**Single Infection**			
*Trichuris*	11	21.6	10.3–32.9
*Ascaris*	9	17.6	7.8–29.4
**Multiple Infection**			
*Trichuris*+*Ascaris*	12	23.5	11.8–35.3
*Trichuris*+Hookworm	4	7.8	2.0–15.7
*Trichuris*+*Ascaris*+Hookworm	1	2.0	0–5.9

### Microbial communities of the indigenous Malaysians

**Figure 1:**
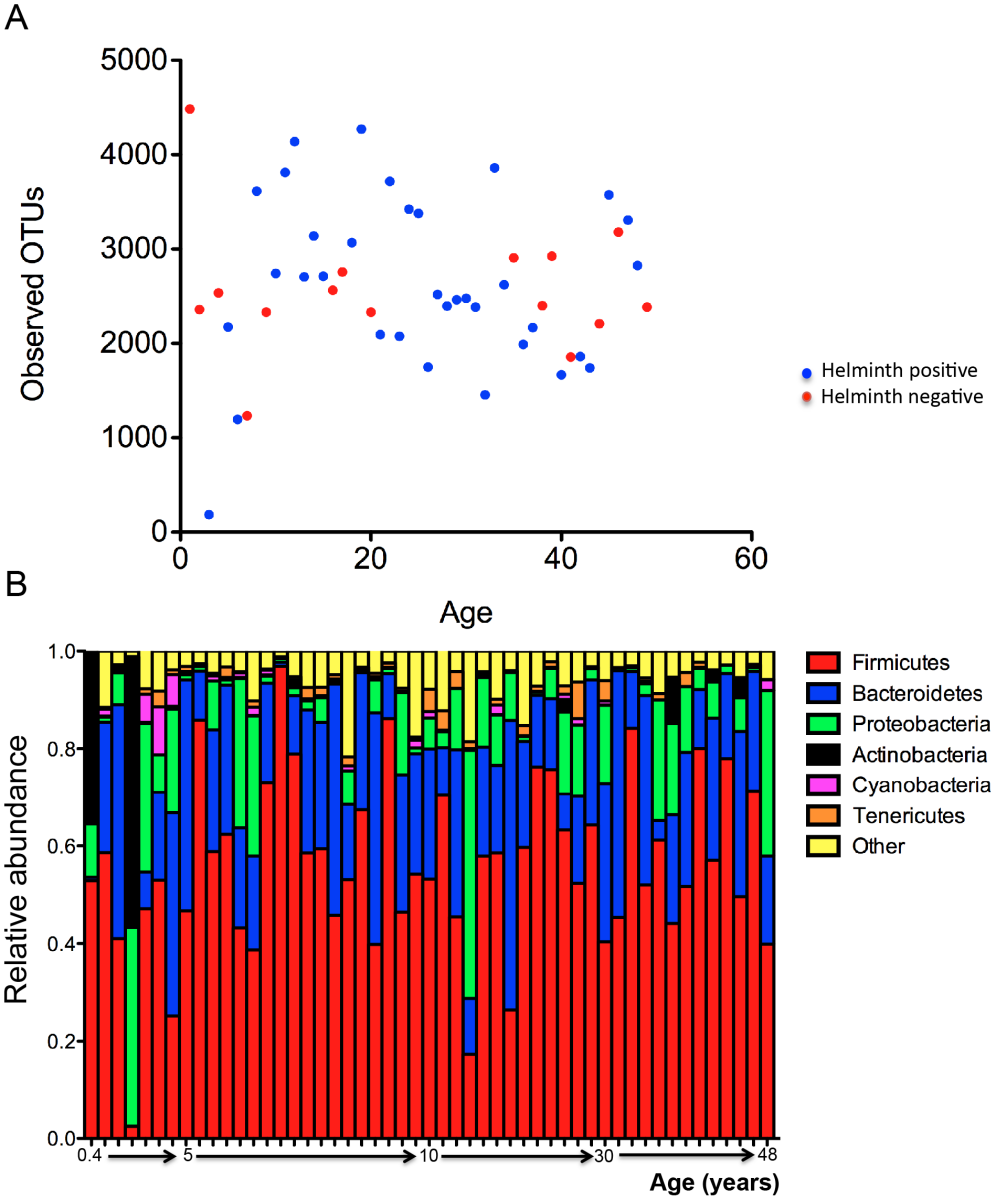
Abundance and diversity in the intestinal microbiome in 51 Malaysian subjects. (**Panel A**) The number of observed OTUs plotted against age for all 51 individual samples. The number of observed OTUs for most samples was between 1500–4000. (**Panel B**) Relative abundance of the top phyla represented across the 51 subjects arranged by increasing age. The abundance patterns were largely similar across the individual subjects, except in two of the younger subjects who had high abundance of Actinobacteria (*Bifidobacterium* sp.) in their stool samples.

The V4 region of bacterial 16S rRNA was PCR-amplified and sequenced on the MiSeq (Illumina) platform from the 51 Malaysian fecal samples. A total of 931,078 quality-filtered sequences were obtained from these samples with an average of 19,002±6,451 (SD) sequences per sample. These reads were clustered into 83,457 unique OTUs with an average of 1,703 OTUs per subject. The number of observed OTUs was similar across the different age groups ranging from 1000–4500 (**Figure 1A**). Averaging across samples, the most abundant phyla were Firmicutes (55.9%), Bacteroidetes (23.5%), and Proteobacteria (10.1%). This pattern was largely similar across the individual microbiota, with the exception that in two younger subjects, age 5 months and 3 years old, in whom we found unusually high abundance of Actinobacteria (35.23 and 55.49%), which was resolved to the genus *Bifidobacterium* (**Figure 1B**).

### Colonization with helminths is associated with increased species richness of the microbiota

**Figure 2:**
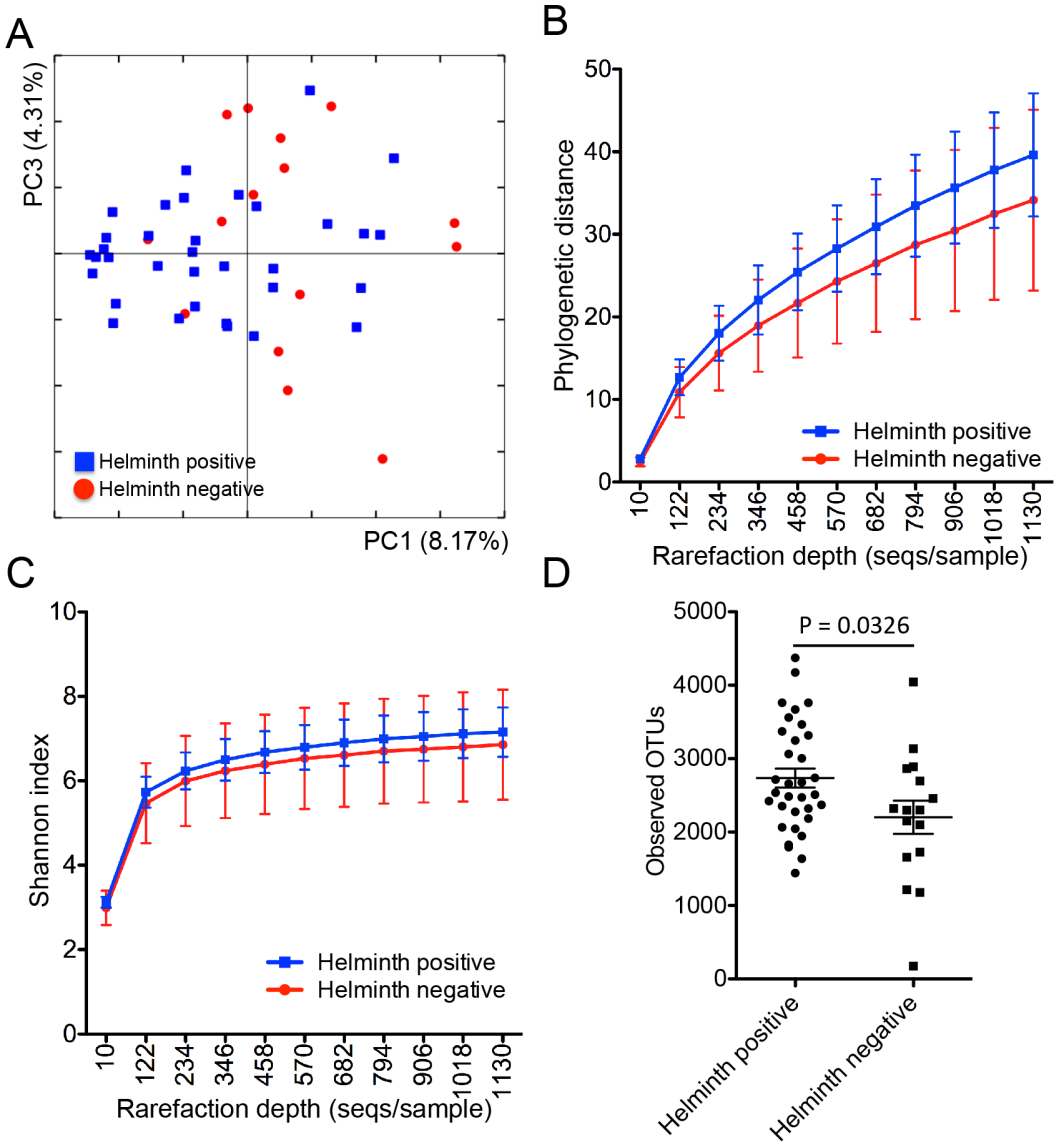
Beta and alpha diversity for the 51 subjects. (**Panel A**) PCoA of the microbial communities in helminth-positive and helminth-negative samples. Clustering of helminth-positive subjects could be observed, which is statistically significant (p = 0.04). Rarefaction curves calculated for phylogenetic distance (**Panel B**) and Shannon index (**Panel C**) demonstrating the higher microbial diversity found among helminth positive subjects. (**Panel D**) Total number of observed OTUs in each individual sample was compared between helminth positive and negative groups.

When subjects were classified based on helminth infection status, the PCoA plot generated from the unweighted UniFrac distance matrices on an uneven OTU table suggested clustering of helminth-positive subjects along the first principle coordinate (PC1), representing 8.17% of intersample variance (**Figure 2A**). This difference in bacterial communities was significant, as determined using the non-parametric statistical test analysis of similarity (ANOSIM), where R = 0.18 (p = 0.04).

Alpha diversity analysis was performed on samples after rarefaction to 1139 sequences/sample (minimum sampling depth). Rarefaction curves generated for the Phylogenetic Distance and Shannon index showed that the helminth-positive subjects demonstrated greater diversity than the helminth-negative subjects (**Figures 2B and 2C**). Two-sample t-test performed on both metrics showed that the helminth-positive subjects had significantly greater species richness (total number of species present) than the helminth-negative subjects (p = 0.04), but the difference in evenness (number of organisms per species) between both groups were insignificant (p = 0.3). The number of observed OTUs was also significantly different (p = 0.0326) between the helminth positive and negative subjects (**Figure 2D**). These results suggest that helminth colonization significantly affects the gut microbiota and may increase the species diversity of the bacterial communities.

### Bacterial taxa populations associated with helminth infected individuals

**Figure 3:**
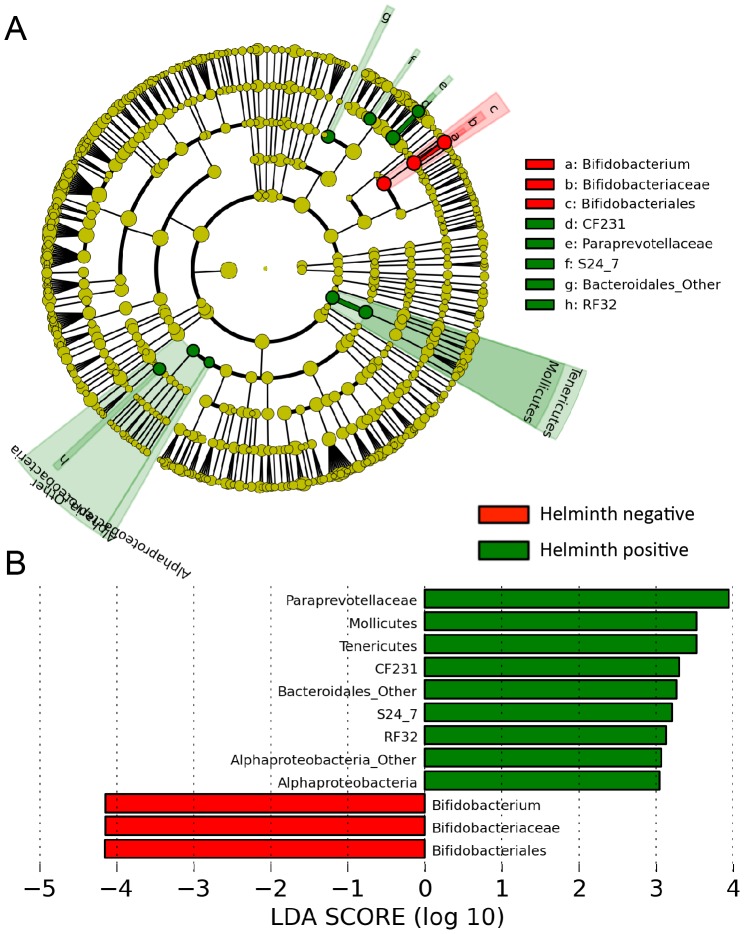
Different abundances of bacterial communities between helminth-positive and negative subjects. With LEfSe for data analysis and visualization key OTUs were identified as differentiating between helminth-positive and helminth-negative fecal samples. (**Panel A**) Bacterial taxa that were differentially abundant in the gut microbiota profiles of helminth positive and helminth negative subjects visualized using a cladogram generated from LEfSe analysis. (**Panel B**) With a log LDA score above 3.00, we found an increased abundance of OTUs contributed by Paraprevotellaceae, Mollicutes, Bacteroidales and Alphaproteobacteria among helminth-positive subjects, while helminth-negative subjects had increased abundance of *Bifidobacterium*.

We next performed a supervised comparison of the microbiota between helminth-positive and helminth-negative subjects by utilizing the LEfSe algorithm to identify taxonomic differences associated with helminth infection status (**Figure 3**). We used a logarithmic LDA score cutoff of 3.0 to identify important taxonomic differences between infected and uninfected individuals. This analysis revealed that helminth-infected individuals have increased abundance sequences representing Paraprevotellaceae, Mollicutes, Bacteroidales, and Alphaproteobacteria. Helminth-negative subjects had an increased abundance of *Bifidobacterium*.

While LEfSe provides an LDA score for class comparisons in order to identify bacterial components that are different between helminth positive and negative individuals through the Kruskal-Wallis (KW) sum-rank test, it may be subject to compositional artifacts and noise common to microbiome datasets. To address these problems, we developed a method for combining centered log-ratio (clr) transformations of the relative abundance data (to circumvent the compositional bias problem), in combination with sparse(to ensure selection of relatively few OTUs) Partial Least Squares Discriminant Analysis (sPLS-DA) to generate predictive models of OTU features (see Methods).

**Figure 4:**
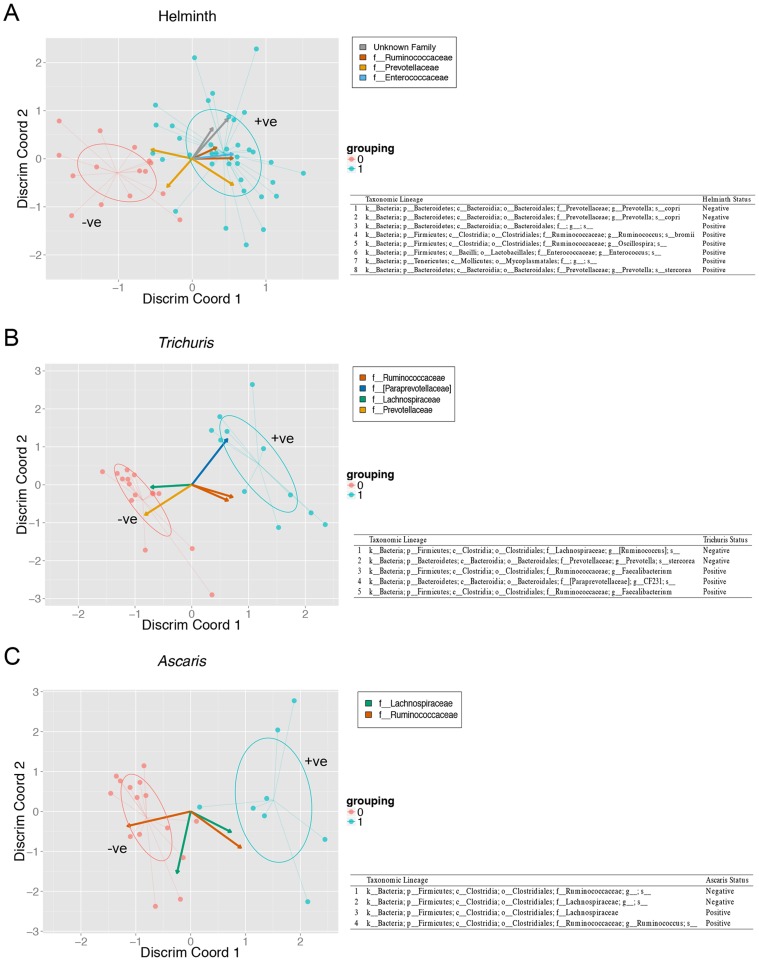
Identification of helminth associated OTUs by centered log-ratio (clr) transformations and sparse Partial Least Squares Discriminant Analysis (sPLS-DA). Supervised sPLS-DA models were first used to identify OTUs associated with helminth infection (**Panel A**), Trichuris alone (**Panel B**) or Ascaris alone (**Panel C**) with statistically significant model coefficients (alpha = 0.05). These OTUs then underwent additional permutation testing by fitting sPLS-DA models to over a thousand randomized datasets. These models were then visualized using biplots of the first two PLS factors.

We first used this approach to identify OTUs associated with overall helminth infection status (**Figure 4A**), and also with the subset of subjects infected with either *Trichuris* alone (**Figure 4B**) or *Ascaris* alone (**Figure 4C**). In our implementation, we perform cross validation analysis to select algorithm parameters, and generate bootstrapped (n = 1000) estimates of model coefficients, selecting only OTUs with statistically significant pseudo p-values (alpha = 0.05). These models were then visualized using biplots of the first two PLS discriminant components (**Figure 4**) using the ggplot2 package [49]. With helminth colonization as a whole, there was an unclassified Bacteroidales associated with infection. However, other families (Lachnospiraceae and Prevotellaceae) had OTUs that are both positively and negatively associated with colonization, which was difficult to interpret. What was more interesting was that *Trichuris* alone was strongly associated with Paraprevotellaceae, consistent with the LEfSe analysis, indicating that *Trichuris* colonization maybe the driving force for this association.

### Alterations to microbial function in helminth colonized individuals

**Figure 5:**
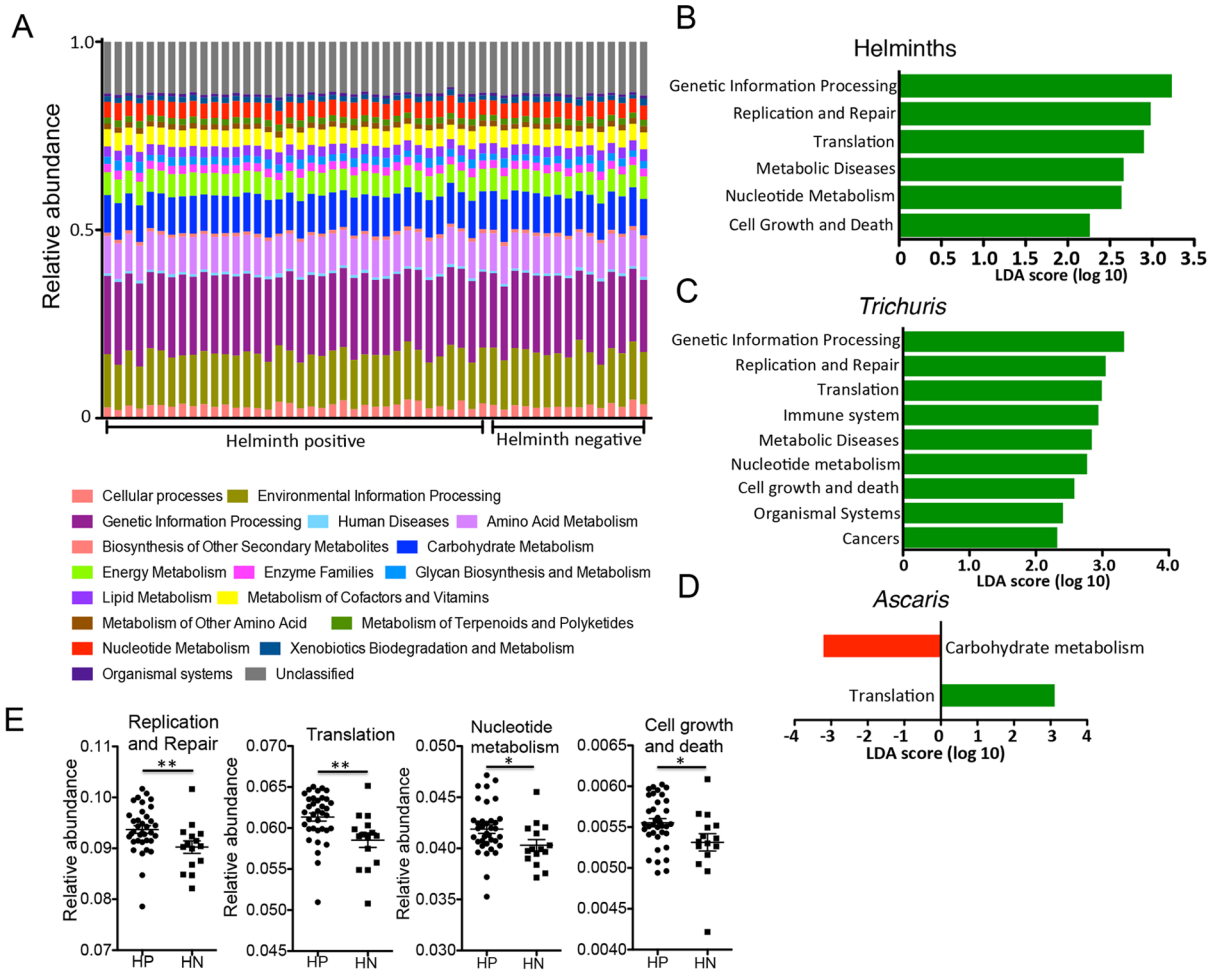
Inferred metagenomic analyses with PICRUSt. (**Panel A**) Relative abundances of KEGG pathways encoded in the gut microbiota of helminth positive and negative indigenous Malaysians. (**Panel B**) Supervised comparison using LEfSe identifies differentially abundant KEGG pathways (Log LDA>2.00) in individuals positive for helminths. No functional pathways were differentially abundant in the helminth negative individuals. (**Panel C**) A similar list of functional pathways was found to be differentially abundant in individuals infected with Trichuris alone. (**Panel D**) Comparison between individuals who were positive for Ascaris only and individuals who were negative for helminths found translational pathways to be differentially abundant in the former (labeled green) and carbohydrate metabolism pathway to be differentially abundant in the latter group (labeled red). (**Panel E**) Genes in the pathways of Replication and repair, Translation, Nucleotide metabolism and Cell growth and death are more abundant in helminth positive individuals.

We next utilized inferred metagenomics by PICRUSt [28] to investigate functional differences in the microbiota of the 51 individuals. As noted above, the relative abundance of different bacterial taxa among the villagers varied considerably between individuals (**Figure 1B**). However, when we assessed the microbial metabolic and functional KEGG pathways of these communities by inferred metagenomics using PICRUSt [28], [29], the pathways were more evenly distributed and consistent between individuals (**Figure 5A**). This stability of metabolic pathways despite variability of microbial taxa was also observed in the Human Microbiome Project [27].

To identify microbial functional pathways that may be altered by helminth infection, we performed supervised comparisons with LEfSe. The gut microbiota of helminth positive individuals have functional pathways more abundant for genetic information processing, particularly pathways for translation, replication and repair (**Figure 5B**). The pathways for nucleotide metabolism, as well as pathways for cell growth and death were also of significantly higher abundance in the microbiota of helminth positive individuals (**Figure 5B**). This effect is largely due to the presence of *Trichuris* worms, since many of the same functional pathways are identified when comparing individuals colonized with *Trichuris* alone with individuals that are helminth negative (**Figure 5C**). At a higher KEGG pathway hierarchy level, there were differences in microbial metabolic pathways between the helminth positive and negative individuals (**Figure S1**). Carbohydrate and xenobiotic metabolism pathways were enriched in the microbiota of helminth negative individuals, while microbiota of helminth positive individuals encoded increased abundance of metabolic pathways involving nucleotides, amino acids, terpenoids and polyketides, novobiocin biosynthesis and for vitamins and co-factors involving one carbon pool by folate. The reduced usage of carbohydrate metabolic pathways is likely to be driven by the presence of *Ascaris* worms, since this pathway was also identified as differentially enriched in helminth negative individuals when performing a comparison with individuals colonized only by *Ascaris* (**Figure 5D**).

### Differences in the gut microbiota of Malaysian and New York City residents

To validate our sampling, sequencing and analysis approaches, we wanted to replicate findings from previous studies describing microbiota differences between developing and developed countries. We compared the microbiota of the 51 individuals from these Malaysian indigenous communities with individuals living in a westernized environment such as the USA. Nineteen fecal samples from healthy adult men in New York City previously analyzed with the same MiSeq sequencing platform was used as this reference group. The combined data set of 51 fecal samples from the Malaysian indigenous communities and 19 fecal samples from New York gave a total of 1,029,891 sequences and 92,701 OTUs.

**Figure 6:**
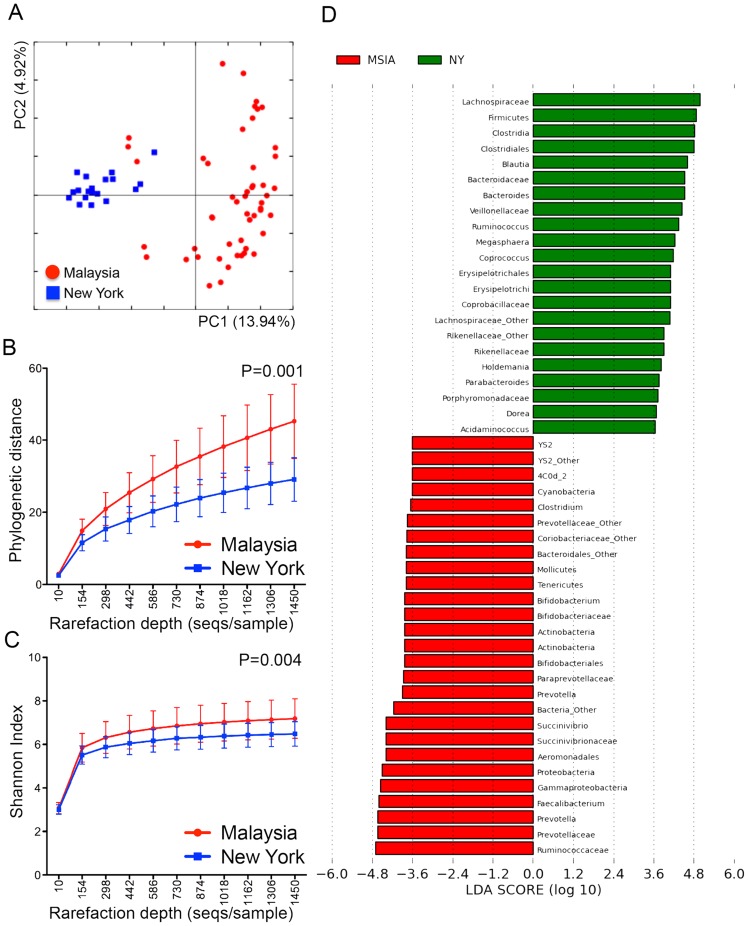
Differences in gut microbiota between New York and Malaysian subjects. (**Panel A**) PCoA plot for beta-diversity patterns of bacterial communities of fecal samples from the Malaysian indigenous community and the New Yorkers. Rarefaction curves plotted for phylogenetic distance (**Panel B**) and Shannon index (**Panel C**) as measures of alpha diversity. Phylogenetic distance and Shannon index were calculated at a rarefaction depth of 1450sequences/sample, and showed significant differences between the Malaysian and New York samples. (**Panel D**) LEfSe analysis of fecal communities showing Firmicutes as the differentially abundant phylum in the New York stool samples (green, NY) while Cyanobacteria, Actinobacteria, Tenericutes, and Gammaproteobacteria were differentially abundant in the Malaysian stool samples (red, MSIA).

By examining unweighted UniFrac distance matrices on an uneven OTU table, these differences translated to distinct clusters visualized on a PCoA plot (**Figure 6A**). The separation between Malaysian and US samples was best seen along PC1, which captured 13.94% of intersample variance. ANOSIM showed that the microbial community differences between the Malaysian and New York subjects were highly significant with R = 0.69 (p = 0.001). This was a much larger difference than the difference between helminth infected and non-infected people in Malaysia.

When we examined alpha diversity (**Figures 6B and 6C**), rarefaction performed on OTU table rarefied to a minimum sampling depth of 1450sequences/sample showed that the stool microbiota of the Malaysian subjects were more diverse than the stool microbiota of the New York subjects. Two-sample t-tests performed for both metrics showed that the differences in microbiota diversity between the Malaysian and New York subjects were highly significant (p = 0.001, p = 0.004).

**Figure 7:**
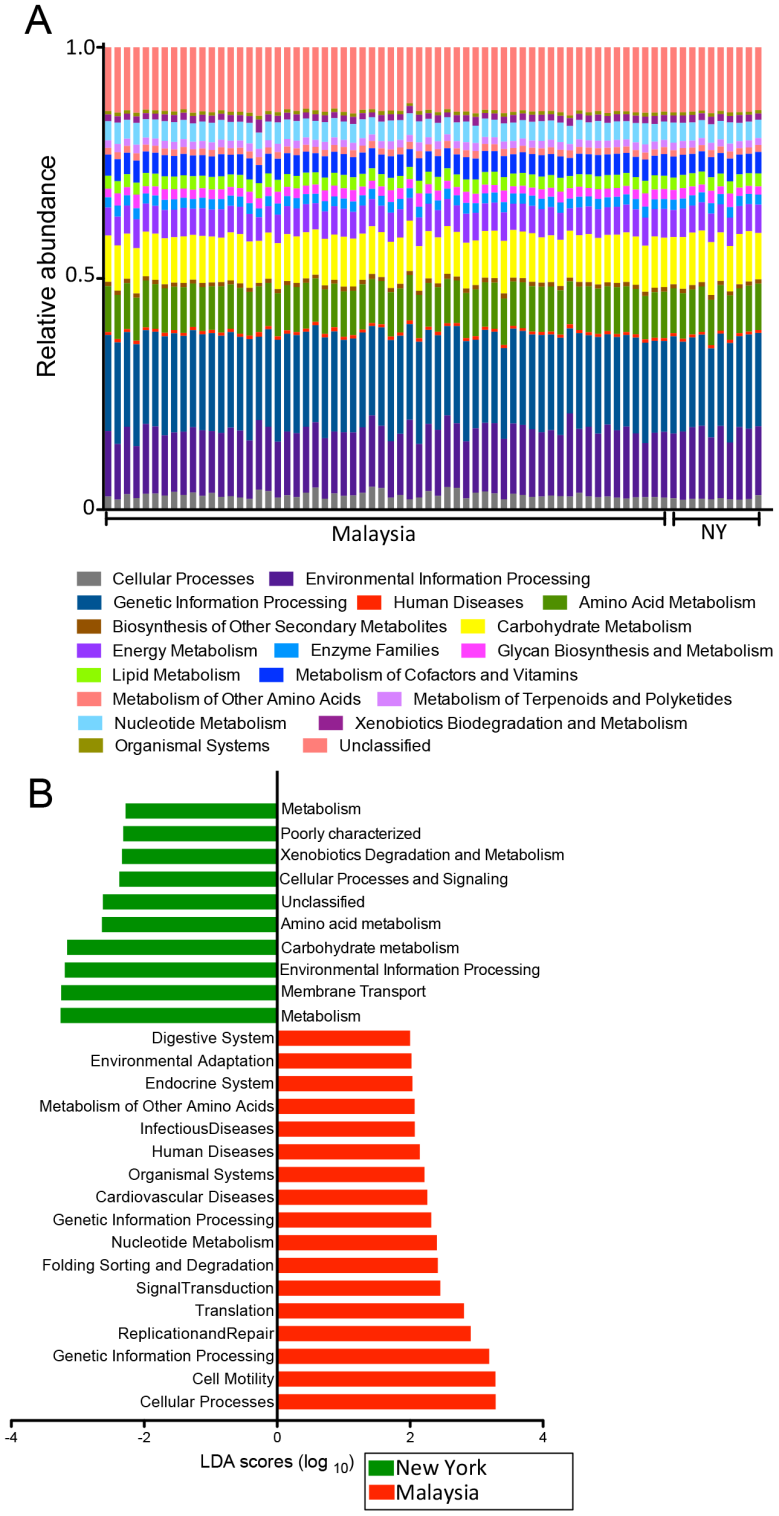
Metagenomic differences between New York and Malaysian subjects. (**Panel A**) Relative abundances of metabolic pathways encoded in the gut microbiota of indigenous Malaysians and New Yorkers. (**Panel B**) Functional divergence between the gut microbiota of indigenous Malaysians and New Yorkers. Supervised comparison identifies differential abundance of specific KEGG pathways using LEfSe (Log LDA>2.00).

A supervised comparison using LEfSe was then performed to statistically define (at log LDA threshold of 3.50) the particular differences in microbial composition between Malaysian and US samples. This confirmed that bacteria from the phylum Firmicutes was more abundant in the New York individuals and the phyla Cyanobacteria, Actinobacteria, Tenericutes and Proteobacteria were more abundant in Malaysian individuals (**Figures S2 and S3**). More specifically, Erysipelotrichi, Ruminococcus, *Bacteroides* and *Blautia* were more abundant for the individuals from New York, while Mollicutes, Gammaproteobacteria, *Faecalibacterium*, *Prevotella* and Ruminococcaceae were more abundant among the Malaysian indigenous people (**Figure 6D**). We then utilized inferred metagenomics by PICRUSt to compare microbial functions between the indigenous Malaysians and New Yorkers (**Figure 7**). Biological pathways encoded were relatively stable (**Figure 7A**), but when we utilized LEfSe to identify specific differences (**Figure 7B**), it was interesting to note that many of the pathways associated with helminth colonization (*e.g.* Genetic information processing, replication and repair, and translation) were also enriched in the indigenous Malaysians vs. New Yorkers comparison (**Figure 7B**). These results suggest that helminth colonization may contribute towards the large differences observed between individuals that live in developing countries and industrialized countries.

## Discussion

In this study, we found that helminth colonization was associated with a significant effect on the gut microbiota of Malaysian indigenous people, with increased diversity and increased abundance of a particular Paraprevotellacae, which may be driven by *Trichuris* infection. However, these differences were much smaller than the differences observed between the Malaysian indigenous people and residents of New York City. This finding is not surprising, since the US residents had much lower bacterial diversity and evenness when compared to the rural Malaysian population, consistent with previous studies comparing rural residents of developing countries with urban residents of developed countries (*e.g.* Bangladesh vs. US [50], Burkina Faso vs. Italy [8], Amazonas and Malawians vs. US [7]). Although the samples from New York that we used to compare with the Malaysians were processed differently did not come from age-matched individuals, they enabled a basis for validating our sampling and sequencing approach. All of these data are consistent with the hypothesis that socioeconomic development is associated with the disappearance of the ancestral microbiota [19], [51].

To our knowledge, this is the first time that the gut microbiota of rural indigenous Malaysians has been investigated. As elsewhere, the phyla Firmicutes and Bacteroidetes dominated the microbiota of most of the fecal samples collected from these Malaysian indigenous communities. Taxa abundances across the different age groups was largely similar, consistent with studies showing that the phylogenetic composition of the human intestinal microbiota evolved to resemble an adult-like configuration within the first three years of life [7]. Since among the 51 subjects from whom we collected the fecal samples, only five subjects were three years old or younger, data was limited for this early period. Nevertheless, the unusually high abundance of *Bifidobacterium* in two of the young subjects is consistent with prior observations of its enrichment in the infant gut [7], [8].

In animal models, the nematode *Heligmosomoides polygyrus* was found to alter the gut microbiota of healthy mice, with increased *Lactobacillaceae* after infection [52]. *Trichuris suis* infection also has an effect on the intestinal microbiota of pigs, with an increase of the mucus colonizing *Mucispirillum* bacteria, and shotgun sequencing showed reduce carbohydrate metabolism, with significant reductions in cellulolytic *Ruminococcus* [53]. There were also significant differences in microbial composition between pigs able to clear adult worms compared to those remaining colonized [36]. Pigs with heavy parasite burden were associated with greater *Campylobacter* abundance [36]. In cattle, infection with the nematode parasite *Ostertagia ostertagi*, which infects the abomasum and may be less sensitive to the mucosal immune responses elicited by these helminths, led to minimal microbiota changes [54]. Helminth infection of macaques with colitis can reverse dysbiosis, restoring communities to resemble those in healthy macaques [22].

Since helminths co-exist in the intestinal tract with the gut microbiota, significant interactions between these organisms are not surprising. *Trichuris muris* eggs utilize the cecal microbiota as environmental cues to enable hatching and the exit of the larvae [55]. Fewer *Heligmosomoides polygyrus* adult worms were recovered from germ-free than conventional mice during infection, associated with increased eosinophilia, granulomata, and thickening of the small intestinal wall [56], indicating that the commensal bacteria may reduce the inflammatory response against the worms, and that *H. polygyrus* requires them to develop appropriately. In summary, there appears to be significant cross-talk between helminths and the gut microbiota.

The increase in bacterial alpha-diversity among helminth-infected individuals could be important, because higher microbiota diversity has generally been associated with better health [57]. This concept of ecosystem stability is supported by both animal models and clinical studies [22], [50], [58]. However, we did not observe differences in community evenness (Figure 2C). The helminth effects on bacterial diversity but not evenness may reflect our small study size. From an ecological perspective, microbial evenness may correlate with community function after stressor induced perturbations [59]. Macaques with colitis had reduced diversity of the mucosal microbiota that was increased following therapeutic helminth infection [22].

However, it is important to note that a recent study on school children in Ecuador did not observe statistically significant effects of *Trichuris trichiura* infection on bacterial composition or microbial diversity of the microbiota [60]. More studies in the future will be needed to determine if these discrepancies are a result of geographical and population differences or a result in the approach used in each study. Indeed the differences we observed between helminth-infected and negative individuals were not dramatic, hence a larger study size in our population is needed to confirm these observations. More importantly, longitudinal studies after anti-helminth therapy of infected individuals would provide a more direct test of the impact of helminths on the gut microbiota. We collected samples from an area where the majority of individuals were infected, indicating heavy helminth exposure. The helminth-negative individuals are likely to be continually exposed to helminths, and some may be infected but not detected through our microscopy-based examinations. One possibility is that past helminth exposure may have already altered the gut microbiota, although the subjects may have been negative at the time of study.

Although the samples from New York that we used to compare with the Malaysians did not come from age-matched individuals, they enabled a basis for validating our sampling and sequencing approach, replicating prior studies comparing developing country rural communities with developed country urban communities [7], [8], [50]. The greater diversity and evenness of the gut microbiota among the Malaysian indigenous community was consistent with prior findings showing that rural Amerindian and Malawian adult populations had significantly more diverse gut microbiota than American adults [7], [8], [50].

Bacteria from the phylum Firmicutes, which was more abundant among the New York subjects, has been linked to obesity [15], [61] and with antibiotic treatment [62]. Findings of increased *Prevotella* among the Malaysian samples and increased *Bacteroides* among the New York subjects were also consistent with previous reports on the *Prevotella*/*Bacteroides* difference between developed and developing countries. This difference has been attributed to dietary differences, with *Bacteroides* associated with diets rich in animal protein, several amino acids, and saturated fats (common in developed countries) and *Prevotella* being associated with carbohydrates, simple sugars, and high fiber diet (common in developing countries) [8], [63].

However, it is important to note that storage conditions for stool samples prior to DNA extraction were different for the Malaysian subjects and VA subjects, which could have uncontrolled effects on bacterial diversity. While stool samples from New York were frozen immediately, samples from Malaysia were stored at 4 degrees for up to a week prior to extraction. We have not experimentally determined that storage conditions do not affect bacterial diversity, however all of the samples from the Malaysian subjects were collected on the same day and DNA was also extracted from all of the samples on the same day. Hence, all of the samples were stored for the same period of time and storage conditions should have had the same effect on all the samples with regards to bacterial diversity. Nonetheless, future studies should evaluate freshly frozen stool samples collected in the field.

The inference of microbial function by the prediction of bacterial metagenome using PICRUSt added another dimension in characterizing the differences of the microbiota between helminth positive and helminth negative individuals. The differences in gene contents of the intestinal bacteria between helminth positive and helminth negative individuals were largely driven by *Trichuris* infection. We have previously suggested that *Trichuris* infection can lead to an increase in mucus production and epithelial cell turnover, consequently reducing the number of bacteria attached to the intestinal wall and restoring the diversity of mucosal bacteria [22]. As such, the higher rate of bacterial cell turnover in the gut of *Trichuris* positive individuals, as reflected by the increased abundance of genetic information processing and cell cycle pathways, could be an effect of the microbial diversity restoration process associated with ongoing *Trichuris* infection. However, since these differences were not observed in other studies [60], it will require further confirmation to better characterize the functional effects of helminth induced microbiota compositional changes.

In conclusion, this study provides a preliminary view of the effects of helminth infection on the human gut microbiota in the indigenous communities of Malaysia. Although differences between helminth-positive and negative subjects are not as substantial as those with urban US residents, greater bacterial diversity appears associated with helminth colonization. While we cannot determine if these are causal relationships, these results will help direct future investigations of the relationship between helminths and the gut microbiota in developing countries. Perhaps in the future, these relationships can be exploited therapeutically for the treatment of autoimmune diseases, since helminths already are being studied in clinical trials.

## Supporting Information

Figure S1 **Functional pathways that were differentially abundant in the gut microbiota profiles of helminth positive individuals and helminth negative individuals (LDA score >0.02) when the predicted metagenome was annotated using a higher KEGG pathway hierarchy level.** A higher number of microbial functional pathways related to metabolism (pathways marked with an asterisk) were noted to be differentially abundant between helminth positive and helminth negative individuals. The intestinal bacteria of helminth positive individuals were enriched with metabolic pathways related to nucleotides, amino acids, terpenoids and polyketides, novobiocin, vitamins and co-factors involving one carbon pool by folate. The intestinal bacteria of helminth negative individuals were enriched with metabolic pathways involving carbohydrate and xenobiotics (chlorocyclohexane and chlorobenzane). Different pathways related to genetic information processing were enriched in both groups of individuals – intestinal bacteria of individuals who were helminth positive had a higher abundance of pathways related to replication and cell growth, while that of the helminth negative individuals had a higher abundance of pathways related to signal transduction.
(TIF)

Figure S2 **Relative contribution for most abundant taxa in fecal samples from New York and Malaysian subjects.** Taxa were resolved to different levels and averaged across samples from the two studied populations. 95.2% of the 16S rRNA gene V4 sequences from the combined Malaysian and New York data sets were constituted by the five major phyla of Firmicutes (59.2%), Bacteroidetes (24.2%), Proteobacteria (8.7%), Actinobacteria (2.1%) and Tenericutes (1.0%). The most pronounced difference in relative abundance was observed for the phylum Firmicutes (68.8% in the New York samples vs. 56.1% in the Malaysian samples), followed by differences in the relative abundance of Proteobacteria (10.5% in the Malaysian samples vs. 3.9% in the New York samples). At the class level, the fecal samples from the New Yorkers had higher relative abundances of Clostridia (63.6% vs. 52.8%), Erysipelotrichi (4.2% vs. 1.8%) and Betaproteobacteria (1.7% vs. 0.5%) while the Malaysian fecal samples had higher relative abundance of Gammaproteobacteria (9.3% vs. 1.6%). At the order level, we found prominent differences in the relative abundances of bacterial taxa that were contributed by the Proteobacteria phylum: Burkholderiales were higher among the New York samples (1.7% vs. 0.4%) while Aeromonodales were higher among the Malaysian samples (5.4% vs. 0.1%). At the family level, the New Yorkers had higher abundances of Lachnospiraceae (Firmicutes) (35.4% vs. 19.1%) and Bacteroideceae (Bacteroidetes) (9.7% vs. 3%) while the Malaysian indigenous community had higher abundances of Ruminococcaceae (Firmicutes) (25.6% vs. 15.7%) and Prevotellaceae (Bacteroidetes) (17.4% vs. 9%). At the genus level, the major Bacteroidetes among the Malaysian subjects was *Prevotella*, in contrast to *Bacteroides* for the New Yorkers. The main Firmicutes in the stool microbiota of the Malaysian samples was *Faecalibacterium* while *Blautia* was found at higher abundance in the New York samples.
(TIF)

Figure S3 **Bacterial taxa that were differentially abundant in the gut microbiota profiles of indigenous Malaysian and New York subjects, visualized using a cladogram generated from LEfSe analysis.** At a log LDA threshold score of 3.50, we found that the gut microbiota profile of the indigenous Malaysian subjects were differentially enriched with bacterial taxa contributed by the phyla Proteobacteria, Actinobacteria, Tenericutes and Cyanobacteria. In contrast, the phylum Firmicutes contributed bacterial taxa that were differentially abundant in the gut microbiota profile of the New York subjects.
(TIF)
