# Correction: Helminth Colonization Is Associated with Increased Diversity of the Gut Microbiota

**DOI:** 10.1371/journal.pntd.0009325

**Published:** 2021-04-07

**Authors:** Soo Ching Lee, Mei San Tang, Yvonne A. L. Lim, Seow Huey Choy, Zachary D. Kurtz, Laura M. Cox, Uma Mahesh Gundra, Ilseung Cho, Richard Bonneau, Martin J. Blaser, Kek Heng Chua, P’ng Loke

Explanation of the Correction provided by the authors:

The authors were recently brought to attention about inconsistencies in the color-coding of helminth positive vs. helminth negative individuals between panels in Figs 1 and 2 of this publication [1]. During the process of responding to these queries, we identified mislabeling in the metadata for the analysis of the alpha diversity metric Observed Operational Taxonomic Units (OTUs), where helminth positive and helminth negative individuals were not matched to the correct values of Observed OTUs. The corrected values along with the associated helminth colonization status are presented here. There is no association between Observed OTUs and age using the corrected values, as described in the original publication (Fig 1A). However, we found that the differences in Observed OTUs between helminth positive and helminth negative individuals were statistically not significant (p = 0.73, Fig 2D, graph 1), which was different from our original report. While there was no mislabeling in the calculations of the alpha diversity metrics Faith’s Phylogenetic Diversity and Shannon index, we have re-visualized the values of these two alpha diversity metrics in similar boxplot formats for comparison. The Phylogenetic Diversity of the gut microbiota between helminth positive and helminth negative individuals was significantly different (p = 0.041, Fig 2D, graph 2), while the Shannon index of the gut microbiota between helminth positive and helminth negative individuals was statistically not significant (p = 0.303, Fig 2D, graph 3), as reported in the original publication. Values for each alpha diversity metric, along with the associated helminth status, are available in S1 File. The original publication has not been corrected online.

Since these corrections constitute an important component of the study, we wish to address several conclusions that have been discussed in the original publication. First, the gut microbiota of helminth positive individuals in this study have higher species richness (measured using Phylogenetic Distance), but there appears to be no differences in the number of distinct OTUs or species evenness (measured using Shannon index). While these inconsistencies between alpha diversity metrics could simply be due to the small sample size, it could reflect the underlying biology of intestinal helminth colonization and gut microbiota, or sensitivity of the Kato-Katz technique to differentiate positive and negative individuals. In a follow up study, we demonstrated a significant reduction in Observed OTUs among individuals treated with deworming medication [2]. Deworming also led to significant alterations in the members of the gut microbiota. The paired analysis performed in the deworming study accounted for inter-individual variations and hence, was better suited for detecting direct diversity changes in the gut microbiota. Finally, we have also deposited the raw sequencing data from this study on Qiita [3] (Study 12138). We thank Lauren Carruthers for bringing these mistakes to our attention. We regret these errors and sincerely apologize for the inconvenience caused to readers.

Fig 1A is incorrect. The authors have provided a corrected Fig 1 here.

**Fig 1:**
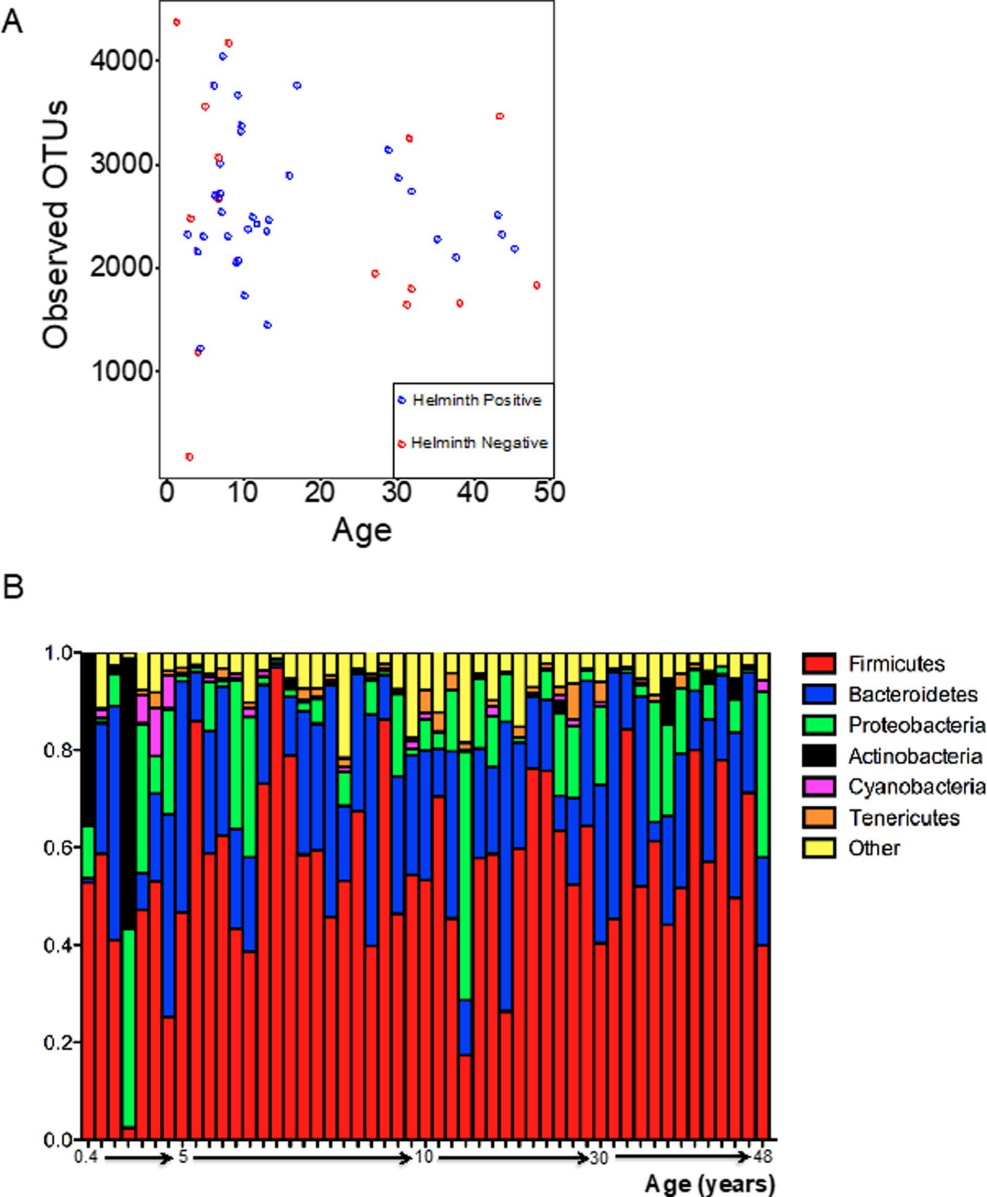
Abundance and diversity in the intestinal microbiome in 51 Malaysian subjects. (**Panel A**) The number of observed OTUs plotted against age for 49 individual samples. The number of observed OTUs for most samples was between 1500–4000. (**Panel B**) Relative abundance of the top phyla represented across the 51 subjects arranged by increasing age. The abundance patterns were largely similar across the individual subjects, except in two of the younger subjects who had high abundance of Actinobacteria (*Bifidobacterium* sp.) in their stool samples.

Fig 2D is incorrect. The authors have provided a corrected Fig 2 here.

**Fig 2:**
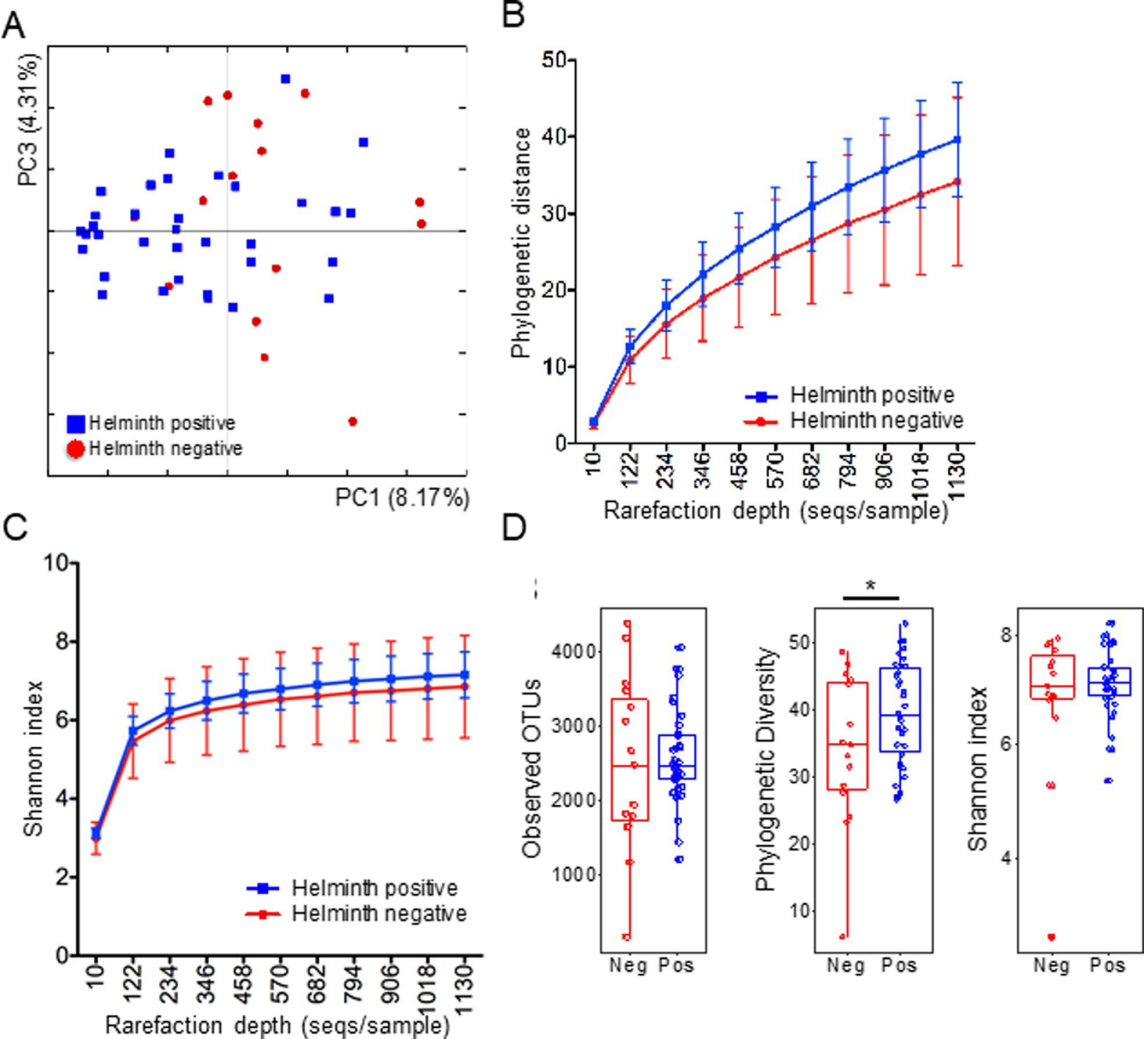
Beta and alpha diversity for the 51 subjects. (**Panel A**) PCoA of the microbial communities in helminth-positive and helminth-negative samples. Clustering of helminth-positive subjects could be observed, which is statistically significant (p  =  0.04). Rarefaction curves calculated for phylogenetic distance (**Panel B**) and Shannon index (**Panel C**) demonstrating the higher microbial diversity found among helminth positive subjects. (**Panel D**) Alpha diversity metrics (observed OTUs, Phylogenetic Diversity and Shannon Index) were compared between helminth positive (n = 34) and negative (n = 15) individuals. Analysis methods are detailed in the original publication. Two individual samples with < 1000 sequencing reads were removed from the alpha diversity analysis. * p < 0.05 by non-parametric two-sample t-test as implemented in QIIME compare_alpha_diversity.py.

## Supporting information

S1 File Supplementary dataset. Values for each alpha diversity metric, along with the associated helminth status.
(TXT)
