# Supplementary figures and images for: Helminth Colonization Is Associated with Increased Diversity of the Gut Microbiota
Source: PLoS Negl Trop Dis. 2014 May 22;8(5):e2880. doi: 10.1371/journal.pntd.0002880 (PMC4031128; doi:10.1371/journal.pntd.0002880)

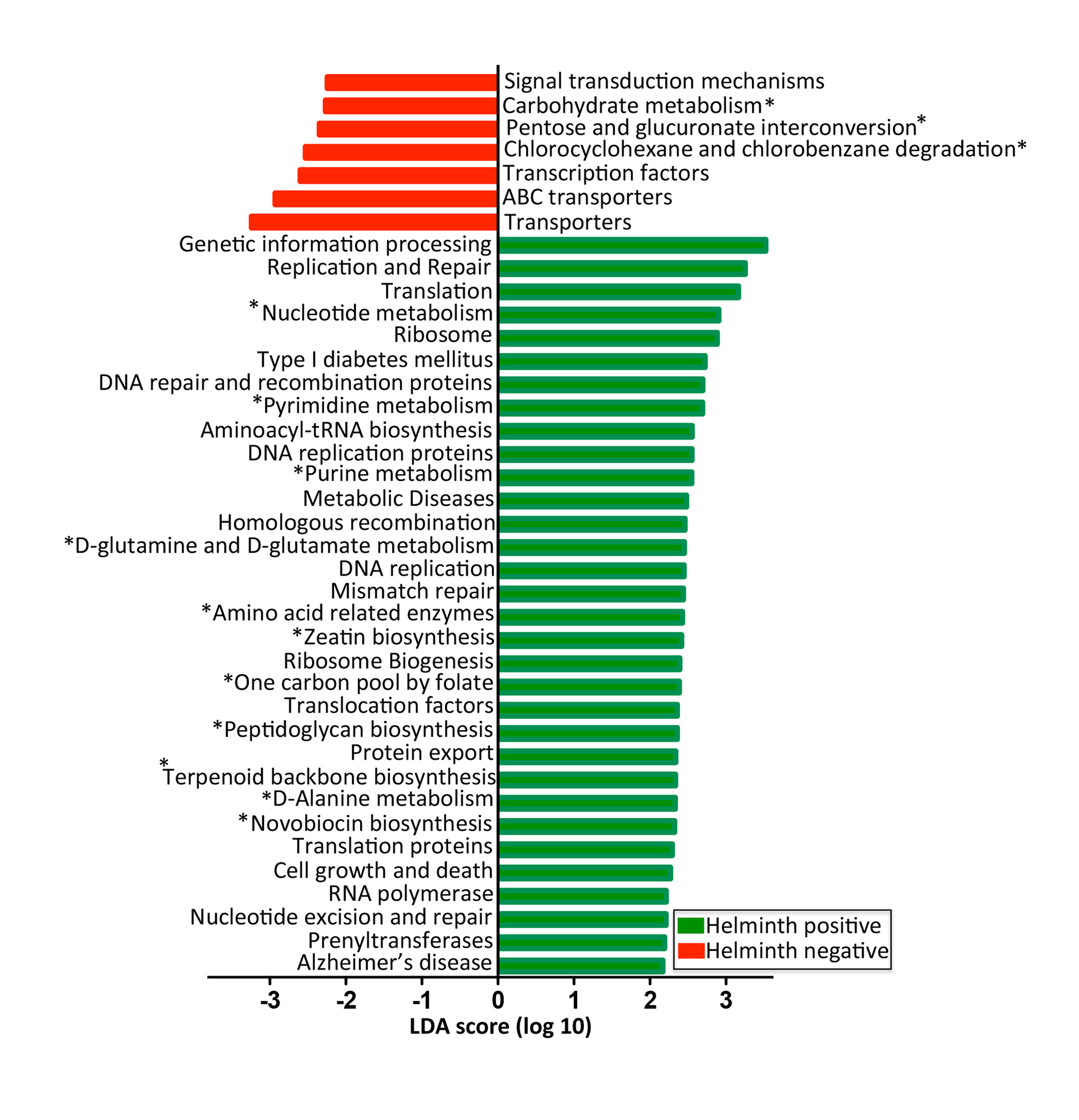

Supplement: Figure S1 — Functional pathways that were differentially abundant in the gut microbiota profiles of helminth positive individuals and helminth negative individuals (LDA score >0.02) when the predicted metagenome was annotated using a higher KEGG pathway hierarchy level. A higher number of microbial functional pathways related to metabolism (pathways marked with an asterisk) were noted to be differentially abundant between helminth positive and helminth negative individuals. The intestinal bacteria of helminth positive individuals were enriched with metabolic pathways related to nucleotides, amino acids, terpenoids and polyketides, novobiocin, vitamins and co-factors involving one carbon pool by folate. The intestinal bacteria of helminth negative individuals were enriched with metabolic pathways involving carbohydrate and xenobiotics (chlorocyclohexane and chlorobenzane). Different pathways related to genetic information processing were enriched in both groups of individuals – intestinal bacteria of individuals who were helminth positive had a higher abundance of pathways related to replication and cell growth, while that of the helminth negative individuals had a higher abundance of pathways related to signal transduction. (TIF) [file pntd.0002880.s001.tif]

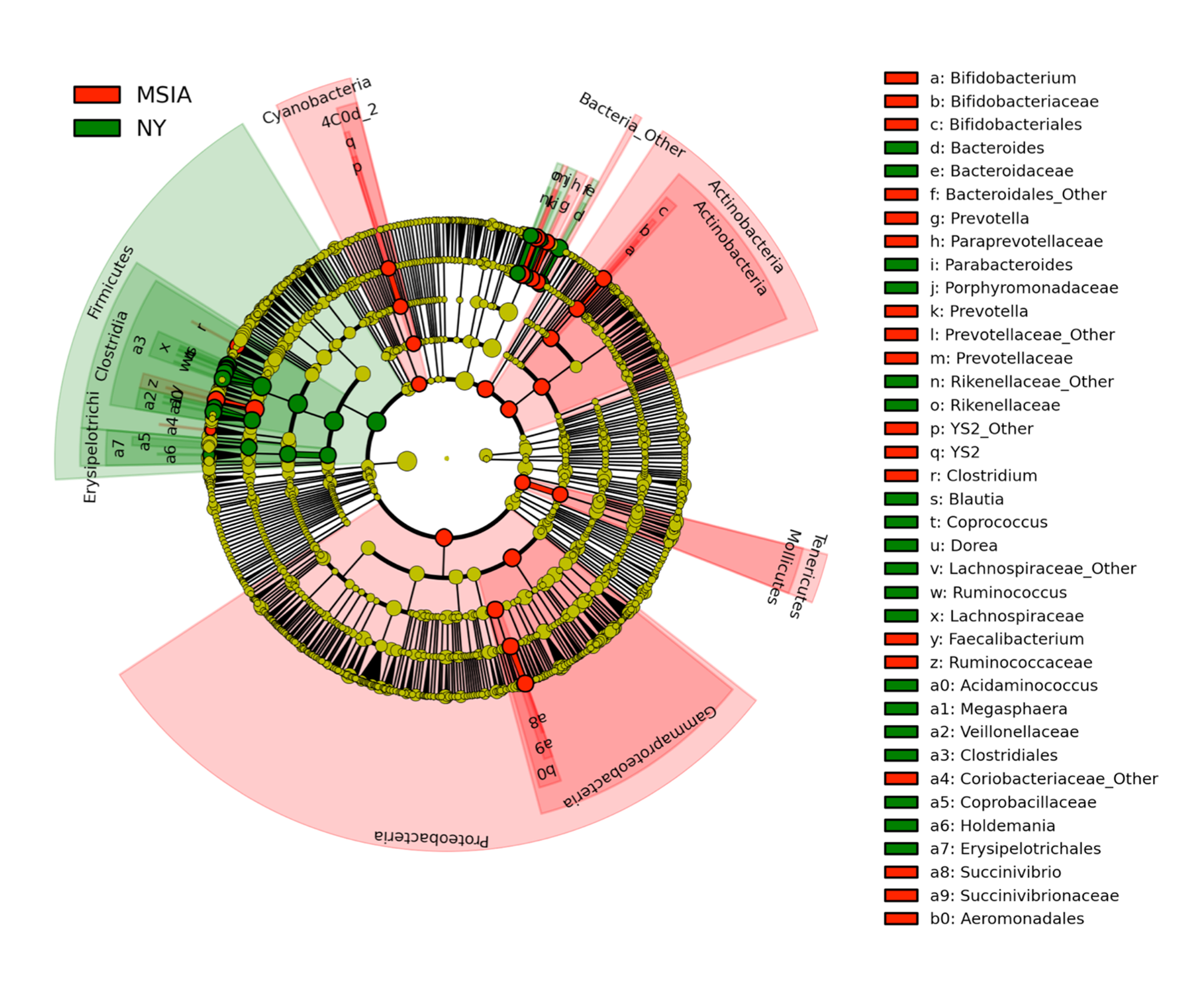

Supplement: Figure S2 — Relative contribution for most abundant taxa in fecal samples from New York and Malaysian subjects. Taxa were resolved to different levels and averaged across samples from the two studied populations. 95.2% of the 16S rRNA gene V4 sequences from the combined Malaysian and New York data sets were constituted by the five major phyla of Firmicutes (59.2%), Bacteroidetes (24.2%), Proteobacteria (8.7%), Actinobacteria (2.1%) and Tenericutes (1.0%). The most pronounced difference in relative abundance was observed for the phylum Firmicutes (68.8% in the New York samples vs. 56.1% in the Malaysian samples), followed by differences in the relative abundance of Proteobacteria (10.5% in the Malaysian samples vs. 3.9% in the New York samples). At the class level, the fecal samples from the New Yorkers had higher relative abundances of Clostridia (63.6% vs. 52.8%), Erysipelotrichi (4.2% vs. 1.8%) and Betaproteobacteria (1.7% vs. 0.5%) while the Malaysian fecal samples had higher relative abundance of Gammaproteobacteria (9.3% vs. 1.6%). At the order level, we found prominent differences in the relative abundances of bacterial taxa that were contributed by the Proteobacteria phylum: Burkholderiales were higher among the New York samples (1.7% vs. 0.4%) while Aeromonodales were higher among the Malaysian samples (5.4% vs. 0.1%). At the family level, the New Yorkers had higher abundances of Lachnospiraceae (Firmicutes) (35.4% vs. 19.1%) and Bacteroideceae (Bacteroidetes) (9.7% vs. 3%) while the Malaysian indigenous community had higher abundances of Ruminococcaceae (Firmicutes) (25.6% vs. 15.7%) and Prevotellaceae (Bacteroidetes) (17.4% vs. 9%). At the genus level, the major Bacteroidetes among the Malaysian subjects was Prevotella, in contrast to Bacteroides for the New Yorkers. The main Firmicutes in the stool microbiota of the Malaysian samples was Faecalibacterium while Blautia was found at higher abundance in the New York samples. (TIF) [file pntd.0002880.s002.tif]

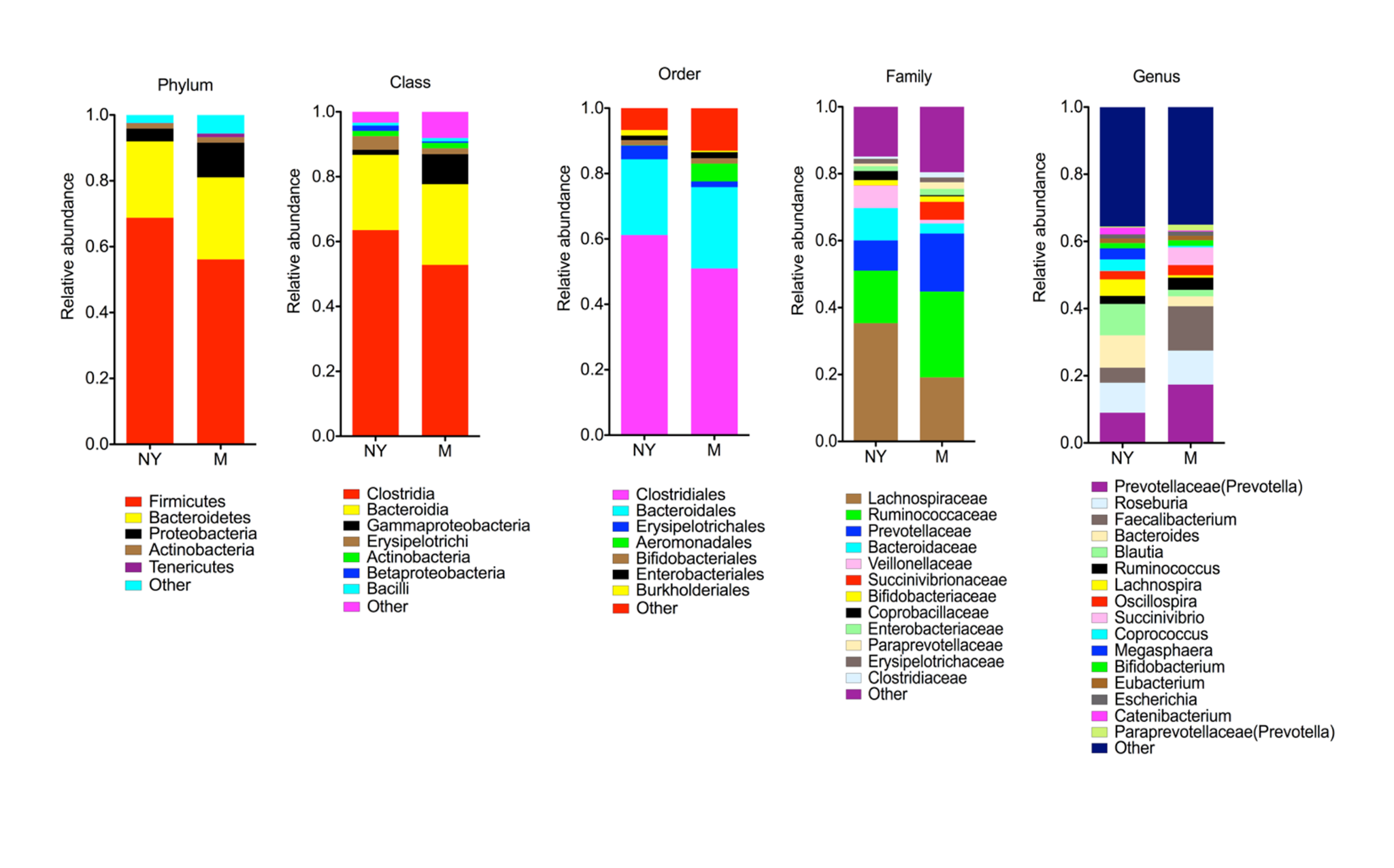

Supplement: Figure S3 — Bacterial taxa that were differentially abundant in the gut microbiota profiles of indigenous Malaysian and New York subjects, visualized using a cladogram generated from LEfSe analysis. At a log LDA threshold score of 3.50, we found that the gut microbiota profile of the indigenous Malaysian subjects were differentially enriched with bacterial taxa contributed by the phyla Proteobacteria, Actinobacteria, Tenericutes and Cyanobacteria. In contrast, the phylum Firmicutes contributed bacterial taxa that were differentially abundant in the gut microbiota profile of the New York subjects. (TIF) [file pntd.0002880.s003.tif]
